# Impact of FDI, industrialization, and education on the environment in Argentina: ARDL approach

**DOI:** 10.1016/j.heliyon.2023.e12872

**Published:** 2023-01-11

**Authors:** Liton Chandra Voumik, Mohammad Ridwan

**Affiliations:** a Department of Economics, Noakhali Science and Technology University, Noakhali, Bangladesh, 3814

**Keywords:** Argentina, ARDL, CO_2_ emission, Education, Industrialization, STIRPAT

## Abstract

**Purpose:**

This study employed the stochastic implications of regression on population, affluence, and technology (STIRPAT) methodology between 1972 and 2021. The main goal of this research is to look at how FDI, population growth, industrialization, and education affect the environment in Argentina.

**Methodology:**

The F-bound test and Johansen cointegration test are employed in this research to determine if there is a co-integration association among the variables. Additionally, the Autoregressive Distributed Lag (ARDL) method is used to examine the short-run and long-run elasticity of the independent variable. This study also incorporated a pairwise Granger causality test to determine the direction of causation between the variables. After that, the study applied several diagnostic and stability tests.

**Findings:**

The empirical evidence demonstrates the presence of a co-integration association among CO_2_ emissions, population, industrialization, and education. The findings indicate that population growth and industrialization harm the environment in Argentina in the long run. In addition, a significant inverse association was obtained between CO_2_ emissions and educational expenditures in the short run.

**Practical implications:**

The existence of STIRPAT suggests that Argentina is capable of achieving sustained economic growth. To achieve the goal, countries must implement appropriate government policies and ensure their implementation. This paper argues strongly for more investment in education, renewable energy, sustainable industrialization, and research and development, all of which are essential for a green economy.

## 1 Introduction

In the 21st century, atmospheric greenhouse gas (GHG) concentrations are a major contributor to climate change. The main drivers of greenhouse gas emissions are human activities like deforestation and burning fossil fuels [1]. Experts estimate that if CO_2_ emissions continue to grow, they will have enormous impacts on global climate change, with possibly disastrous implications for every sector of society [2]. Lowering carbon dioxide emissions and conserving ecological integrity have become worldwide priorities for sustaining economic growth and mitigating climate change [3]. Additionally, the Sustainable Development Goals (SDGs) that the United Nations (UN) has set for 2030 emphasize the urgent need for technical innovation, broad-based sustainable economic development, and access to renewable energy (SDGs 7, 8, 9, and 13). Several countries in Latin America have already seen the significant effects of climate change [4]. Argentina is one of the numerous emerging market nations that have been criticized for overwhelmingly encouraging gross domestic product (GDP) without regard for the environment [5]. In addition to being Latin America's third-largest greenhouse gas emitter (after Brazil and Mexico), Argentina was selected for research as an example since there is existing theoretical and empirical research on the issue. Additionally, Argentina has pledged to reduce carbon emissions by 30% by 2030 as part of its Nationally Determined Contribution (NDC). On the other side, Argentina is confronted with significant environmental challenges. To gain a balance between climate change policies and sustainable development objectives, policymakers must understand Argentina's susceptibility to climate change. The trade-off between environmental damage and development is the most challenging part of attaining these two objectives at once. Therefore, sustainable development and enhanced ecological sustainability (emission reduction) are competing goals. Examining Argentina's environmental factors can help provide an answer to an important issue that comes to the fore: how might Argentina minimize CO_2_ emissions? Environmental degradation will unavoidably emerge if the linkage between energy resources and economic expansion plans cannot be regulated. These problems are especially prevalent in emerging nations like Argentina, where it is important to simultaneously encourage economic expansion, energy security, industrialization, and ecological sustainability. Moreover, the Intergovernmental Panel on Climate Change (IPCC) claims that developing market economies prioritize economic development above environmental protection, as seen by the expansion of their economies while producing about 76.6% of the world's GHG emissions, mainly CO_2_ emissions [6]. Argentina is known as one of the developing market economies that has consistently implemented strategies to promote economic growth while neglecting environmental pollution [7]. Argentina is Latin America's biggest emerging economy and has industrialized its economy to increase its pace of economic development. As a result, despite economic progress, the country's carbon dioxide emission statistics have deteriorated. For example, Argentina's yearly per capita GDP and carbon dioxide emissions both increased at the same time, by about 22% and 8%, respectively, between 2000 and 2018 [8]. In 2020, the contribution of the industrial sector to the GDP would increase to 23.33% from 22.01% in 2016 [9]. Argentina's industrialization initiatives, which have constantly supported the country's attempts to industrialize its economy to expedite economic growth, might be attributed to these emerging trends. Accordingly, throughout the period from 2000 to 2020, the country's indicator of trade openness increased by about eight percentage points [8]. This index is calculated using the GDP's percentage share of exports and imports. Furthermore, the connection between FDI and environmental degradation in Latin American countries has been extensively explored [[10], [11], [12]] Argentina's development economy benchmark is heavily influenced by peripheral factors such as foreign direct investment (FDI), which increases GHG emissions and hence impacts environmental value. Argentina's development strategy is analogous to global practice, in which governments are concerned about sustaining the existing pace and level of economic growth. According to the World Bank [8], the yearly percentage shares of FDI inflows to the nation's GDP almost quadrupled, which is consistent with the upward trend of GDP. The potential negative environmental effects of FDI in Argentina may be explained by the fact that FDI has likely increased the country's energy consumption, which is largely met by fossil fuels. In Argentina, nonrenewable fossil fuels account for over 72% of all electrical production. Therefore, rising energy demand, FDI inflows, and CO_2_ emissions show that Argentina's FDI and industrialization policies are not environmentally sound.

Education may have an impact on climate change by fostering “green” innovation. Several studies have connected human capital and skills to a country's ability to adhere to environmental standards and lessen pollution [13–15]. It is generally recognized how important skills are for innovation, especially the development and adoption of new technologies. However, increasing educational spending promotes green economic development and reduces carbon emissions by raising awareness [16]. The Argentine government prioritizes education spending to boost economic growth, productivity, and personal and social development. In 2020, Argentina's educational spending as a percentage of GDP increased from 18.1% to 8.1%. The amount spent on education in Argentina as a percentage of GDP increased by 39.2% between 2010 and 2020 [8]. Argentina spent about $31 billion on education in 2020, with $5 billion going to private schools and $26 billion to government-run institutions [8]. Thus, it is important to examine the connection between Argentina's increasing education spending and ecological sustainability.

This investigation focused on Argentina's economic development and environmental quality within the STIRPAT framework. Here, CO_2_ emissions are a surrogate for environmental degradation, while FDI is a proxy for economic affluence. This analysis employed the ARDL model to determine the long- and short-run nexus and causal relations among FDI, industrialization, education, population, and CO_2_ emissions. Where industrialization and education expenditure are used as proxies for technology in the STIRPAT model.

The following are the principal purposes of this study.

1. To ascertain the effect on the environment within the STIRPAT framework in Argentina.
2. To explore the short- and long-term linkages between CO_2_ emissions and FDI, population, industrialization, and education in Argentina.
3. To detect the causality between CO_2_ emissions and FDI, population, industrialization, and education in Argentina.

The primary implications of this paper are: 1. Theoretically and conceptually, variable selection in Argentina is justifiable; 2. This analysis used the most recent and exhaustive data available; 3. The findings have been acquired by using proper econometric techniques, which were then analyzed using a variety of diagnostic tests; 4. This will aid comprehension of the requirement for more effective research and development initiatives; 5. The study results are appropriate and consistent with the prevailing theories and national context; 6. This research will assist policymakers in building effective strategies in Argentina and throughout the world.

Following is the structure of the remaining parts: Section 2 is a comprehensive assessment of the literature review. This study's data and econometric approach are summarized in Section 3. Section 4 and 5 demonstrates and discusses the econometric analysis findings and discussion, along with relevant sources. Sections 6 and 7, respectively, explain the conclusion and the policy recommendations.

## 2 Literature review

Numerous empirical and theoretical studies on the nexus between FDI, industrialization, education, and CO_2_ emissions have been conducted. These studies are conducted on a national or international scale. Albulescu et al. [17] evaluated the linkage between FDI, income, and ecosystem integrity in 14 Latin American economies, including Argentina. The study utilized the panel quantile regression method and observed that there is no remarkable influence of FDI on environmental deterioration. Wang et al. [18] used the ARDL method for newly industrialized countries to examine how FDI affected CO_2_ emissions. The study's results demonstrated that FDI significantly increases CO_2_ emissions. Accordingly, Farooq [19] experimented to ascertain the influence of foreign assistance and FDI on CO_2_ in Asian economies. In doing so, the study employed generalized least squares (GLS), fully modified ordinary least squares (FMOLS), and the generalized method of moments (GMM) estimation approach for regression. The results implied that the flow of FDI boosts CO_2_ emissions due to increased industrial activity. Similarly, Gong et al. [20] investigated the nonlinear dynamic FDI-emissions nexus in “One Belt, One Road” economies. To ascertain the nonlinear link between the variables, the researchers utilized the BDS approach and the Granger causality test. The threshold vector error correction model (TVECM) and threshold vector autoregressive (TVAR) are also employed. The findings of the investigation revealed a causal interaction between FDI and CO_2_ emissions. Furthermore, some evidence suggests that FDI has a long-term significant positive impact on CO2 [[21], [22], [23], [24]]. In contrast, Haug and Ucal (2019) observed that FDI inflows have no remarkable long-term effect on CO_2_ emissions per capita. In contrast, Mujtaba et al. [25] assessed the connection between industrialization and CO_2_ emissions in SSA economies.

Adebayo et al. [26] performed a study utilizing ARDL to determine the factors mitigating human-induced CO_2_ emissions in Argentina. According to the study, higher economic growth resulting from more industrialization has negative impacts on the environment and releases CO_2_. Mentel et al. [27] investigated the correlation between industrialization and CO_2_ emissions in Sub-Saharan Africa (SSA). Employing the GMM method, the researchers determined that the proportion of industry to GDP had a substantial favorable effect on CO_2_ emissions. Accordingly, Azam et al. [28] undertook research to determine the causality between trade, industrialization, urbanization, and CO_2_ in OPEC nations. The paper applied the fixed effect estimator favored by the Hausman test and the robust least squares approach to validate the analysis. The study also employed the D-H Granger causality method to find out the causal linkage. The outcomes of the research reveal that rapid industrialization increases environmental pollution, and the findings of Granger's causality show that a two-way causality exists between industrialization and carbon dioxide emissions. Similarly, Siddique [29] investigated the nexus between energy use, industrialization, and environmental deterioration in South Asia. The augmented mean group (AMG), the correlated effects mean group (CCEMG) analysis, the Westerlund cointegration test, and the D-H causality test was applied throughout this work. The empirical findings reveal that long-run cointegration exists between industrialization and environmental pollution; bi-directional causality also exists between those variables. Moreover, some studies have observed a remarkably favorable impact of industrialization on CO_2_ emissions [[30], [31], [32], [33]]. Elfaki et al. [34], on the other hand, found that the elasticity of industrialization has a negative relationship with environmental degradation and reduces CO2 emissions.

Education is considered the key element of human capital [35]. Increased education spending will result in a greater accumulation of human capital due to higher personal productivity [36]. According to existing literature, human capital investment has been found to have various advantages. Human capital contributes favorably to economic growth and increased labor productivity [37,38], and it is associated with many positive consequences, such as increased democratic involvement, reduced rates of violence, and health improvements [39]. Several studies have shown that human capital adversely affects energy use, which ultimately improves the environmental quality [40]. The well-educated population of the USA benefits from favorable environmental circumstances as a result of its wealth and structural transformation, according to Goetz et al. [41]. In a similar framework, Bano et al. [42] investigated the impact of human resource development on CO_2_ and found that increases in human capital led to lower carbon emissions over time. Short-term analysis, however, reveals no correlation between them. Another study by Lin and Raza [43] discovered that greater levels of human resources have been demonstrated to increase short-term carbon emissions while simultaneously reducing long-term emissions. Increased human capital reduces the ecological footprint by improving the short- and long-term environmental consequences.

Xin et al. [44] examined the influence of unemployment and education on CO_2_ emissions using an ARDL model approach for the Chinese economy. The outcomes of the empirical study demonstrated that average years of schooling and literacy rates curb CO_2_ emissions over time. Mehmood [14] investigated the linkages between renewable energy, female workers, education spending, and CO_2_ emissions in Bangladesh, India, Pakistan, and Sri Lanka. Utilizing the CS-ARDL method, the study demonstrated that higher education expenditure lowers environmental pollution in the long run. Moreover, Eyuboglu and Uzar [45] explored the impacts of higher education on emissions of CO_2_ in Turkey. In line with the study's findings, higher education has a negative association with CO_2_ emissions.

After reviewing the existing literature, we found that there is no existing literature on the impact of FDI, industrialization, and education on the environment in Argentina. The research added unit root tests, cointegration and ARDL bound tests, the ARDL model, Granger causality, and several diagnostic tests. In the aforementioned literature, FDI helps host economies grow by increasing capacity via new investments in greenfield projects or existing operations, which causes carbon emissions in the production units engaged in the advancement of the new capacity or expansion of the prevailing capacity. On the contrary, several studies have found that FDI does not have any substantial influence on CO_2_ emissions. The existing literature demonstrated that rapid industrialization involved higher fossil fuel combustion and harmed the environment, while spending on education played a remarkable role in mitigating CO_2_ emissions. Consequently, it is essential to determine the effect of FDI, industrialization, and education on CO_2_ emissions in Argentina.

The following are the study's contributions to the existing body of knowledge: First, this research evaluates for the first time the impacts of population, FDI, industrialization, and education on environmental deterioration in Argentina. Second, the study included potential factors to investigate the connection between population, FDI, industrialization, education, and carbon emissions that had been neglected in previous research. This experiment will assist policymakers in managing the growing vulnerability caused by climate fluctuations. Third, this study uses the ARDL model with causality and cointegration approaches. The ARDL method provides estimates of both long- and short-run coefficients. This approach produces more accurate findings that facilitate the creation of short- and long-term policy initiatives that promote environmental sustainability. As a result of the sophisticated econometric technique, the results of this research are more helpful for policymakers than those of earlier empirical studies.

## 3 Methodology and data

### 3.1 Theoretical framework and STIRPAT model

The IPAT model is one of the models that can be utilized to evaluate the influence of economic activity on the environment and the amount of energy used. The model uses a random effects regression to evaluate the environmental pressure brought on by demographic variables, wealth, and technological advancement [46]. Equation (1) can be used to depict this.

(1) I=PAT

Where I symbolizes influence, P represents the population, A stands for affluence, and T technology aspects, respectively. The hypothesis cannot be tested because the known values of certain model components dictate the value of the missing term [47]. The STIRPAT model was suggested by Dietz & Rosa [48] to address this shortcoming.

Equation (2) can be used to represent the model.

(2) $$I_{t}=\alpha P_{t}^{b}A_{t\;}^{c}T_{t}^{d}\;\varepsilon_{t}$$

Equation (3) is the result of applying the logarithm (ln) transformation to the non-linear equation (2).

(3) $$\ln\left(I_{t}\right)=\alpha +bln\left(P_{t}\right)+cln\left(A_{t}\right)+dln\left(T_{t}\right)+\varepsilon$$

Here, the coefficients term is denoted by letters a, b, c, and d, the error term is represented by the symbol ε, and the subscripts t indicate time in the proper sequence. The IPAT model enables the analysis of the environmental impact of various substances (CO_2_ emissions, for instance).

Equation (4) is the extended form of previous equations.

(4) $$\ln\;{CO}_{2t}=\beta +\alpha_{1}{lnPOP}_{t}+\alpha_{2}lnFDIt+\alpha_{3}{lnINDUS}_{t}+\alpha_{4}{lnEDU}_{t}+\psi_{t}$$

Where t represents the period, β is the intercept, $\psi_{t}$ is a residual or error term with a normal distribution, and $\alpha_{i}\text{,}$ (i = 1, …, …, 4) are the population, FDI, industrialization, and education coefficients, accordingly.

### 3.2 Empirical model and method of estimation

In this inquiry, methods for inferential data analysis were used. It includes diagnostic tests as well as the ARDL bound test, Granger causality test, and unit root test.

#### 3.2.1 Unit root tests

Since time series data were employed in this study, it is crucial to ascertain if the variables are stationary or not. This investigation also utilized the PP (Phillips &Perron [49]), IPS (Im et al., [50]), and KPSS (Kwiatkowski et al., [51]) unit root tests to observe the stationarity.

#### 3.2.2 Autoregressive Distributed Lag model

This study studied the long-term relationship between Argentina's per capita GDP and spending on research and innovation using the ARDL bound co-integration approach after determining the order of integration of the variables. The ARDL model surpasses OLS, VECM, and VAR models in short- and long-term estimations because of its self-defined lag length structure. The ARDL model offers several advantages over competing options since it is not implemented in the same manner as the conventional cointegration approach [52]. Firstly, it is not compatible with high-order variables or I(2), but it is compatible with mixed [I(0) or I(1)] variables and I(0), I(1), fractionally integrated variables [53]. Second, although traditional co-integration approaches need a large sample, the ARDL limits method may be used with a relatively small sample (N < 50). Because the long-run model generates unbiased estimates, it can address serial correlation issues, omitted variables, and endogeneity concerns. ARDL bounds co-integration also has the advantage of combining long- and short-term events into a single, manageable equation [54]. For assessing the long-term relationship between the variables, each significance level has two distinct sets of crucial values. All regressors are supposed to be I(0) in the lower limit critical value set, and all regressors are assumed to be I(1) in the upper limit critical value set. When the projected F-statistic exceeds the upper limit of significance, it is feasible to conclude that the variables used in this analysis are co-integrated. However, if the predicted F-statistic is less than the lower limit of significance, the null hypothesis must be accepted. This demonstrates that the study's variables are not co-integrated. If the estimated F value is between the lower and upper limit critical values, no inferences can be made regarding the co-integration status of the variables employed in this analysis.

An econometric model revealing the STIRPAT model and the impact of current education expenditure on CO_2_ emissions has been built based on existing research. Thus, the following is the extended ARDL model, according to Adeleye et al. [55]:

(5) $$\ln\;{CO}_{2}=\omega_{0i}+\sum_{i=1}^{p}\alpha_{i}\;\ln\;{CO}_{2_{t-i}}+\sum_{i=0}^{q}\beta_{i}{lnPOP}_{t-i}+\sum_{i=0}^{r}\gamma_{i}{lnFDI}_{t-i}+\sum_{i=0}^{s}\delta_{i}{lnINDUS}_{t-i}+\sum_{i=0}^{t}\gamma_{i}{lnEDU}_{t-i}++\sum_{i=0}^{u}\theta_{i}p_{t-i}+\varepsilon_{t}$$

The constant term in Equation (5) is ${\;\beta }_{i}$, and the parameters are $\gamma_{i}\text{,}$ $\delta_{i}$, and ${\;\theta }_{i}$. The optimal lag order is q, and the explained and explanatory variables are expected to be co-integrated or purely I (1). Here, t refers to time, and $\varepsilon_{t}$ denoted the white noise error term.

In this study, error correction representations with the following specifications are used to analyze the event of long- and short-run dynamics:

(6) $$\Delta \ln\;{CO}_{2}=\mu_{01}+\lambda \left({ⱳ}_{1}{lnCO}_{2_{t-i}}+{ⱳ}_{2}{lnPOP}_{t-i}+w_{3}{lnFDI}_{t-i}+{ⱳ}_{4}{lnINDUS}_{t-i}+{ⱳ}_{5}{lnEDU}_{t-i}\right)+\sum_{i=1}^{p}{ⱳ}_{i}\;\ln\;{CO}_{2_{t-i}}+\sum_{i=0}^{q}\beta_{i}{lnPOP}_{t-i}+\sum_{i=0}^{q}\gamma_{i}{lnFDI}_{t-i}+\sum_{i=0}^{q}\delta_{i}{lnINDUS}_{t-i}+\varepsilon_{1t}$$

The null hypothesis [H_0_: ⱳ_1_ = ⱳ_2_ = ⱳ_3_ = ⱳ_4_ = ⱳ_5_ = 0] was opposed to the alternative hypothesis ${[H}_{0}:\;{ⱳ}_{1}\neq {ⱳ}_{2}\neq {ⱳ}_{3}\neq {ⱳ}_{4}\neq {ⱳ}_{5}]$ that the variables included in this analysis are interconnected over the long term.

Comparing the results of the F-test to the specified critical values validated the supposition that there is a long-term link among the variables examined in this research [52] (Pesaran et al., 2001). In Equation (6), λ stands for the speed of adjustment coefficient, which, to have the long-run equilibrium, must be statistically significant, negative, and smaller than one.

#### 3.2.3 Diagnostics test

This study used the Durbin-Watson autocorrelation test (Durbin and Watson [56]), the Breusch-Godfrey serial correlation LM test (Breusch [57]; Godfrey [58]), the White homoscedasticity test (White [59]), the ARCH heteroscedasticity test (Bera and Higgins [60]), the Jarque-Bera normality test (Jarque and Bera [61]), and the Ramsey RESET (Ramsey [62]) specification test to verify the estimated ARDL model. These analyses were performed to check the validity of the data. The predicted model's stability was also verified using the CUSUM and CUSUM square tests [63].

#### 3.2.4 Pairwise granger causality test

According to Granger causality, including the lagged first variable in the test dataset can substantially improve the contingent prediction for the second variable. The study used the pairwise Granger causality test to explore whether or not the variables had a short-run causal link. The causal linkage between X_t_ and Y_t_ can be written as Equation (7):

(7) $$E\left(Y_{t+h}|J_{t,}X_{t}\right)=E\left(Y_{t+h}|J_{t}\right)$$

Where J_t_ reflects the data sets obtained from the Xt and Yt observations made in the past up to that point in time (t). Using the error correction coefficients, this study confirmed the existence and path of Granger causality [64] over the long run between the variables.

### 3.3 Data

The variables in this research have a time range of 1972–2021. Definitions of variables, frequency, and sources are shown in Table 1. The data is annual time series data.

**Table 1:** Variables, frequencies, and data sources.

Variable	Signifier	Description	Source
CO_2_ emission	lnCO_2_	CO2 emissions (kt)	WDI [65]
Population	lnPOP	Population, total	
Foreign direct investment	lnFDI	Foreign direct investment (inflows)	
Industrialization	lnINDUS	Industry (including construction), value added (constant 2015 US$)	
Education	lnEDU	Gov't spending on education, total (% of GDP)	

Table 2 displays log-transformed summaries of the aforementioned variables. Log transformation was used for a more precise measurement of the dependent variable.

**Table 2:** Summary statistics.

Variables	N	mean	sd	min	Max
lnCO_2_	30	11.87	0.198	11.52	12.13
lnFDI	48	21.36	1.748	16.70	23.90
lnPOP	50	17.36	0.183	17.02	17.64
lnINDUS	50	25.32	0.218	24.96	25.69
lnEDU	40	1.097	0.565	0.0451	1.754

## 4 Result and discussion

### 4.1 Unit root test results

For any empirical investigation, variables must be stationary to avoid spurious results. All five variables of this study are subjected to KPSS, PP, and IPS. The unit root null hypothesis cannot be rejected if the test statistic for these tests is insignificant. The result is represented in Table 3.

(a) AIC and SIC have been used to determine the optimal lag time. (b) In addition, an intercept and a trending phrase are added in every unit root test. (c) 1%, 5%, and 10% levels of significance are symbolized by ***, **, *.

**Table 3:** Stationarity tests (Unit root).

Variable	IPS		PP		KPSS		Remark
	At Level	First Diff.	At Level	First Diff.	At Level	First Diff.	
lnCO_2_	−0.055	−5.522***	−1.491	−6.993***	−1.431	−6.993***	Stationary at I(1)
lnPOP	2.452	−3.531***	0.935	−7.693***	0.939	−7.733***	Stationary at I(1)
lnFDI	2.195	−3.380***	1.834	−6.319***	1.811	−7.319***	Stationary at I(1)
lnINDUS	−5.518***		−4.485**		−4.491**		Stationary at I(0)
lnEDU	−0.052	−6.512***	−0.7254	−6.919***	−0.7121	−6.921***	Stationary at I(1)

Based on the data in Table 3, our sample group includes both the I(0) and I(1) series. Results from all three analyses corroborated previous results on CO_2_ emissions, FDI inflows, population growth, industrialization, and education. All variables except industrialization are stationary at the first difference, or I(1). At level or I(0), industrialization is stationary. At the second difference, or I (2), none of the variables are stationary.

#### 4.1.1 Johansen cointegration test

In Table 4a, the Trace statistics are smaller than the critical value (5%). Also, the null hypothesis here is no-cointegration will be rejected if the trace and max-eigenvalue are more than the critical value (5%). So, the research accepted the null hypothesis that there is one cointegration exists and which indicates that the variables are cointegrated in the long run.

**Table 4a:** Johansen cointegration test.

Rank	Trace Statistic	5% Critical Value	Max-Eigen statistic	5% Critical Value
0	61.5214	68.487	24.0654	31.412
1	41.023**	47.024	22.249***	26.124
2	21.032	28.248	14.286	19.224
3	12.241	14.542	8.472	14.174
4	0.254	3.124	0.246	3.725

#### 4.1.2 Bound cointegration test

This study used an ARDL bounds test strategy to look for evidence of co-integration between the variables. Table 4b shows the results.

**Table 4b:** ARDL bound test.

	Test statistics		Value	K
	F		10.265	4
Critical Bounds		Significance level		
		10%	5%	1%
	I(0)	3.28	4.49	5.85
	I(1)	4.3	4.8	6.3

The findings indicate that the null hypothesis of no co-integration is rejected at the 1% significance level. The value of the F test statistic exceeds critical values. Therefore, it may be said that the variables in the model have particular co-integrating relationships. In this research, the long-term driving factors are FDI, industrialization, education, and population, and when a typical stochastic shock hits the system, they move first. The following conclusion implies that CO_2_ emissions change in response to changes in these variables.

### 4.2 ARDL and ECM results

In this study, we examined the long-term association and short-term connection that develop after co-integration forms using the ARDL representation. The result is represented in Table 5.

**Table 5:** ARDL (Short run, long run, and adjusted term).

VARIABLES	ADJ	LR	SR
lnFDI		−0.00254(0.00933)	
lnPOP		1.177*** (0.219)	
lnINDUS		0.399**(0.126)	
lnEDU		0.153(0.0791)	
L.lnCO_2_	−1.133**(0.461)		
D.lnFDI			0.00030(0.0077)
D.lnPOP			7.108(5.797)
D.lnINDUS			0.368*(0.192)
D.lnEDU			−0.099*(0.087)
Constant	−21.78*(9.237)		
R-squared	0.962		

Standard errors in parentheses.

***p < 0.01, **p < 0.05, *p < 0.1.

1%, 5%, and 10% levels of significance are symbolized by ***, **, *. The symbol Δ represents the difference operator.

Source: The authors' calculations.

Table 5 presents the long-run estimates of ARDL. The coefficient of FDI is negative, and the value is −0.00254. The coefficient value is insignificant and shows that there is no long-run cointegration with CO_2_ emission. Accordingly, at a 1% level of significance, the population coefficient demonstrated a substantial positive correlation with CO_2_ emissions. It indicates that a 1% rise in the population will increase CO_2_ emissions by 1.177%. Thus, population growth is a significant cause of CO_2_ emissions in Argentina, as people rely on fossil fuels to drive their increasingly mechanized daily routines. The demand for coal, oil, gas and other fuels that are extracted from the earth continues to be the supply of energy for the population [66,67]. When these fuels are burned, sufficient CO_2_ is emitted into the environment. The findings of this study support previous research showing that increased population growth increases the quantity of CO_2_ emissions released into the environment [25,43,68,69]. The finding contradicts Voumik et al. [70] findings in the Bangladesh contest.

Additionally, the ARDL model's long-run outcomes exhibit that industrialization and CO_2_ emissions are positively correlated. At the 5% level of significance, this correlation between industrialization and CO_2_ emissions is statistically significant. It shows that an increase in industrialization of 1% will also raise CO_2_ emissions by 0.399%. Thus, industrialization has a significant harmful impact on the environment in Argentina. In 2021, the industry sector directly emitted 18% of total CO_2_ emissions in Argentina and became one of the biggest sources of CO_2_ emissions [71]. When fossil fuels are used on-site to generate heat, electricity, or chemical processes, manufacturing, refining, food manufacturing, and other such industrial operations all contribute to CO2 emissions. The finding is in line with the other studies indicating that industrialization has a significant positive contribution to emissions of carbon dioxide [31,69,72].

The results of the ARDL estimates show that education spending has an impact on CO_2_ emissions in the long run, but it is insignificant. Additionally, the short-run ARDL estimates are included in Table 5. FDI, population, and industrialization all have substantial positive relationships with CO_2_ emissions, but all coefficients are insignificant. Only spending on education can minimize CO2 emissions in the short run. A negative education coefficient (−0.099) indicates that, at a 10% significance level, a 1% rise (fall) in education will result in a 0.099% reduction (rise) in CO_2_ emissions. Moreover, for the selected variables, the L.lnCO_2_ coefficient is negative, and there is an annual convergence of 1.133% between short-run and long-run equilibrium.

### 4.3 Granger causality test results

Table 6 shows that FDI granger-causes CO_2_ emission at a 10% level of significance, but CO_2_ does not granger-cause FDI, implying that there is a unidirectional influence running from FDI to CO_2_ emission. On the other hand, the null hypothesis that the population does not Granger cause CO_2_ emissions is rejected at a significance level of 5%. Similarly, the null hypothesis that industrialization does not affect CO_2_ emissions through Granger causation is also rejected at the same level of significance. CO2 emissions cause education, with significant results at 5% of the critical value. Thus, the unidirectional causal link between CO2 emissions and education prevails.

**Table 6:** Granger causality test results.

Pairwise Granger Causality Tests		
**Null Hypothesis:**	**F-Statistic**	**Prob.**
lnFDI ⇏ lnCO_2_	2.96558	0.0715
lnCO_2_ ⇏lnFDI	0.8697	0.4324
lnPOP ⇏lnCO_2_	2.13102	0.034
lnCO_2_ ⇏ lnPOP	0.31079	0.3218
lnINDUS ⇏ lnCO_2_	3.40755	0.027
lnCO_2_ ⇏ lnINDUS	0.05348	0.948
lnEDU ⇏lnCO_2_	0.37371	0.6944
lnCO_2_ ⇏ lnEDU	5.94338	0.0126

Overall, the Granger causality test demonstrates that there is a one-way causal linkage between FDI to CO_2_ emission, population to CO_2_ emission, industrialization to CO_2_ emission, and CO_2_ emission to education at different levels of significance.

### 4.4 Diagnostic tests results

The last issue addressed is the ARDL-error correction model's goodness of fit. Stability and diagnostic tests were conducted for this purpose.

(a) AIC and SIC have been used to determine the optimal lag time. (b) 1%, 5%, and 10% levels of significance are symbolized by ***, **, *.

The model's validity, predictability, and effectiveness are examined using ARDL diagnostic tests, as indicated in Table 7. The BG -LM test indicates that the value of Chi-square is not significant and that the null hypothesis cannot be rejected, indicating that there is no issue with serial correlation. The outcomes of the Whites & ARCH test exhibits that there are no heteroscedasticity issues in the model. To ascertain if the model is free from specification error and normally distributed, the Jarque-Bera and Ramsey RESET tests for model specification and normality are also used. The result of the estimates shows that the value of coefficients is insignificant and the null hypothesis should be rejected indicating that the model is correctly specified and normally distributed.

**Table 7:** Diagnostic tests results.

Statistics test	Statistics	*P*-Value
Breusch-Godfrey LM test for autocorrelation	Chi^2^ (ꭓ^2^) = 0 .359	0.5423
White test for homoscedasticity	ꭓ^2^ = 34.00	0.4157
B–P test for heteroskedasticity	ꭓ^2^ = 0.427	0.3587
Normality test (J-B)	ꭓ^2^ = 2.512	0.4158
LM test for ARCH	ꭓ^2^ = 0.025	0.7845
Ramsey RESET test	F test = 1.78	0.8475
Adjusted R^2^	0.456	

### 4.5 CUSUM and CUSUM square tests

**Fig. 1:**
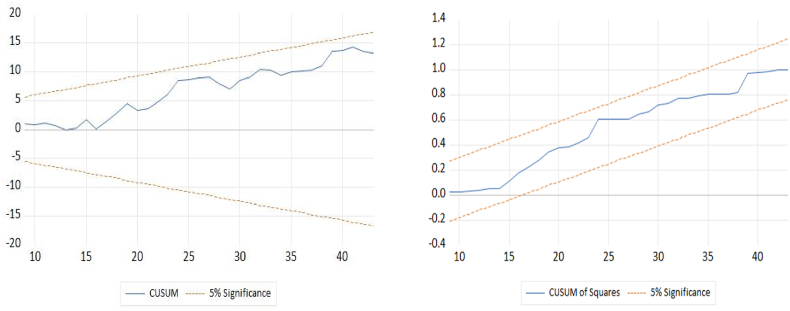
CUSUM and CUSUM square.

In this study, stability is evaluated using the CUSUM and CUSUM square as shown in Fig. 1. If the blue line in the CUSUM and CUSUM square plots continues inside the red border lines at the 5% significance level, then the parameters of the study are stable; if it moves outside of those boundaries, then they are not stable [63]. So, it can conclude that the model used in this study passes the stability tests at the 5% level of significance since the blue line in both the CUSUM and CUSUM square tests falls inside the confines of the red lines.

## 5 Discussion

The impact of FDI is detrimental but insignificant in this research. In most countries, FDI is one of the most pressing environmental concerns. FDI causes environmental issues when some developed countries move their polluting farms and industries to Argentina to avoid rules regarding that production in that developed country. This is a form of getting away with banned operations and productions in one's country by placing those production lines in a country that has not yet banned them. As a rising economy in South America, Argentina should appreciate green FDI and investment in their country. because FDI is one of their main economic pillars. Similarly, the population shows an unfavorable impact on the environment as well. Most of Argentina's cities are overpopulated, and Argentina is one of the most urbanized countries in the world. It's no secret that the country has suffered numerous problems due to its overwhelming population. People are often unaware of the various anti-environmental practices that are seen as the norm in most countries. Hence, they continue to contribute to pollution and environmental degradation without being aware. Excess population means a country needs to produce more, consume more, and engage in more economic activities. All are directly related to CO_2_ emissions. Argentina is a growing country that is facing rapid industrialization. This means the number of production factories and related plants has greatly increased. All these factors are infamous sources of pollution and CO_2_ emissions. Hence, industrialization is affecting the environment, and necessary policies should be adopted to counter that issue. In the short run, education can only minimize environmental degradation in Argentina. The educated generation understands the importance of environmental protection and sustainable development. Also, educated people convince and encourage others not to harm the environment. Education is one of the main tools for improving the environment. Therefore, education is crucial in lowering CO_2_ emissions in Argentina since it has a beneficial impact on the environment by raising residents' levels of social responsibility and, thus, reducing pollution emissions. The study's findings are supported by relevant literature [44,45,73]. On the other hand, in the long run, education's impact is positive but insignificant for Argentina.

## 6 Conclusion

From 1971 to 2021, this study looked at how FDI, industrialization, and education impacted CO_2_ emissions in Argentina. The KPSS, PP, and IPS unit root tests were carried out to validate the hypothesis of stationarity. All variables, except industrialization, were found to be stationary after taking into account the first differences. Because of mixed-order stationary (I(0) and I(1)), the research applied the ARDL method. The benefit of the ARDL method is that it shows short-run, long-run, and adjusted-term findings. The variables are cointegrated, according to the ARDL bound and traditional cointegration tests. In the ARDL findings, CO_2_ emissions are strongly influenced by industrialization and population in the long run. FDI, population, and industrialization have a detrimental impact on the environment, but the impact of FDI is insignificant. Government expenditure on education can only minimize CO2 emissions, and it is significant in the short run. In the short run, the only impact of education is negative and significant. Additionally, the Granger causality test was used to determine the presence of a causal relationship. Granger's causality result suggests that there is a one-way causality between population and CO_2_ emissions, industrialization and CO_2_ emissions, FDI and CO_2_ emissions, and CO_2_ emissions and education. Applying CUSUM and CUSUM square, the paper discovered that the model's predicted coefficients are consistent.

## 7 Policy recommendation

According to the findings presented above, the research recommends adhering to the policies.

• Even though the effects of FDI on the environment are extremely minimal and insignificant, Argentina ought to be conscious of the issue. Inflows of FDI are substantial due to Argentina's attractiveness as an investment destination and the size of its domestic market. Argentina, like many other developing nations, needs to be careful about how it handles foreign direct investment. When it comes to FDI, Argentina should favor environmentally friendly FDI and reject pollution-haven FDI or FDI that causes environmental damage. Because most multinational corporations do not respect domestic laws protecting the environment, they care only about output and profits, which leads to more pollution in the environment. As they have foreign investment, they have enough funds to create an eco-friendly investment that will minimize pollution.
• Even though Argentina has a large land area and a low population density, the country's rapidly growing population is a problem for the degradation of the environment. Loss of biodiversity, deforestation, air and water pollution, and increased pressure on arable land are just some of the environmental stresses linked to population growth. These stresses are exacerbated by the enormous amount of consumption, use of natural resources, increase in economic activities, and production of waste. Consequently, Argentina needs to exercise some sort of population control. Likewise, it is crucial to educate the populace so that they are environmentally conscious. Increase awareness about the environment among people through many advertising activities. The private organization should also arrange some programs to inform them about the environment and explain the negative consequences of deforestation.
• A major contributor to Argentina's rising CO_2_ emissions is the country's burgeoning industrial sector. Most current industrialization is unguided and unsustainable because it lacks both environmental regulations and sustainable planning. To avoid damaging the natural environment and causing deforestation, it is crucial to carefully select the location of industrial facilities. For Argentina's industrialization to succeed, the country must prioritize recycling, proper treatment of industrial waste, and resource cleaning. In this context, having stringent industrial laws and making sure they are enforced is very important.
• Education spending has the greatest potential to reduce ecological degradation in the short run. Because an informed citizen can recognize the importance of addressing sustainability, environmental diversity, and pollution challenges, To maintain a sustainable environment, a generation must be educated to the point where they can comprehend environmental policies and act swiftly. Thus, Argentina needs to boost its investment in education and literacy. Education expenditure and educational institutions should be increased so that a sufficient number of people get the benefit of education. An educated population knows and realizes the need for a healthy environment. So, they protect it from harm and make people aware. There are lots of indigenous people in Argentina. Also, a lot of slum people live in Buenos Aires, Córdoba, Rosario, and other cities. Both indigenous and slum people are poor. The Argentine government should take initiatives so that education is free for low-income and poor people.

## Author contribution statement

Liton Chandra Voumik: Performed the experiments; Contributed reagents, materials, analysis tools or data; Wrote the paper.

Mohammad Ridwan: Conceived and designed the experiments; Performed the experiments; Analyzed and interpreted the data; Wrote the paper.

## Funding statement

This research did not receive any specific grant from funding agencies in the public, commercial, or not-for-profit sectors.

## Consent for publication

N/A.

## Data availability statement

Data will be made available on request.

## Declaration of competing interest

The authors declare no conflict of interest.
